# Precision medicine in the real-world setting. Clinical activity of talazoparib in a woman with hormone receptor-positive HER2-negative metastatic breast cancer with pathogenic mutation in somatic BRCA2

**DOI:** 10.3332/ecancer.2022.1448

**Published:** 2022-09-26

**Authors:** Dana Narváez, Federico Waisberg, Tomás Soulé, Martín Angel, Luisina Bruno, Maria Romina Girotti, Carmen Pupareli, Matías Chacón, Fernando E Petracci

**Affiliations:** 1 Alexander Fleming Institute,Cramer 1180, C.P. 1426, Ciudad Autónoma de Buenos Aires, Buenos Aires, Argentina; 2 Translational Immuno-Oncology Laboratory, Institute of Biology and Experimental Medicine (IBYME), Vuelta de Obligado 2490, Ciudad Autónoma de Buenos Aires, Buenos Aires, Argentina; a https://orcid.org/0000-0002-1463-8887

**Keywords:** breast cancer, somatic mutation, BRCA, PARPi, ESCAT scale

## Abstract

**Background:**

Next-generation sequencing (NGS) has proven to be a key implementation to understanding biological pathways involved in cancer. In daily practice, the identification of somatic and germline mutations has allowed physicians to gather relevant information to make therapeutic decisions and benefit patients. Importantly, somatic mutations provide targeted opportunities for treatment and reveal resistance mechanisms to understand patients’ tumour evolution. Scanty data in clinical trials and in a real-world setting is available regarding the utility of poly(ADP-ribose) polymerase inhibitors in pathogenic or likely-pathogenic somatic breast cancer gene 1/2 (BRCA1/2) mutations and/or germline or somatic Homologous Recombination-Related Gene mutations in advanced breast cancer (ABC).

**Case report:**

Here we report a real-life case of a 47-year-old postmenopausal woman with hormone receptor-positive (HR-positive) Epidermal growth factor receptor 2 (HER2)-negative metastatic BC that had poor response to classic therapeutic strategies for HR+/HER2− ABC. At this point, the possibility of using NGS to guide the treatment was decided in a Molecular Tumour Board (MTB), and the patient had a major response to talazoparib targeting a non-germline BRCA2 mutation.

**Conclusion:**

Undoubtedly, more information regarding the cost effectiveness of NGS is needed to develop adequate reimbursement policies for this technology. It should be highlighted that the generalisation of MTBs and the implementation of molecular screening programmes are greatly needed in our region to gain more knowledge of somatic mutations implicated in the Hispanic and Latin-American population with BC diagnosis. Recently presented results of randomised studies may support the evaluation of somatic mutations with NGS to find targeted therapies for ABC patients.

## Introduction

Approximately 5%–10% of all patients diagnosed with breast cancer (BC) have germline breast cancer gene 1/2 (BRCA1/2) mutations. These proteins play an essential role in cell survival by integrating a complex mechanism of DNA double-strand damage restoration in homologous recombination repair. Four mutations in these genes are highly penetrant and increase the lifetime risk of BC by up to 70% in patients with BRCA1 mutations and by 40%–70% in patients with BRCA2 mutations.

Targeted therapy has been recently explored in patients with these germline mutations, including DNA-damaging agents, such as poly(ADP-ribose) polymerase inhibitors (PARPi), enzymes involved in single-strand DNA strand break repair.

BRCA1/2 are only found as a somatic mutation in 10% of advanced BC (ABC) patients. These somatic BRCA mutations are under-recognised and represent today a missed opportunity for additional targeted therapy. Olaparib and talazoparib are currently approved for patients with germline BRCA1/2 mutations and ABC based on two pivotal Phase III clinical trials.

The experience with PARPi for patients with somatic BRCA1/2 mutations was obtained in small Phase II studies, and current clinical information in real-world experiences is scarce. Nonetheless, the routine evaluation of somatic homologous recombination deficiency (HRD) alterations is not currently recommended by international guidelines [12].

We present a real-life case report on the clinical utility of PARPi in a patient with somatic mutations of BRCA2, for HR+/Her2− ABC and we will discuss our perspective on multigenetic panels for patients with this entity.

## Overview of the case

**Figure 1.**
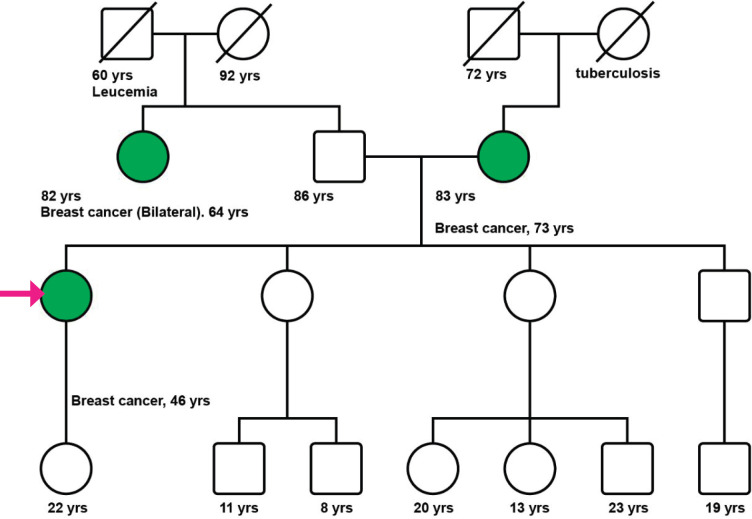
Patient’s pedigree. International nomenclature has been incorporated in the figure.

A 47-year-old postmenopausal woman with a familial history of mother with BC at the age of 73 years (Figure 1), and paternal aunt with synchronous bilateral BC, was diagnosed with primary right BC, hormone receptor-positive (HR+), HER2-negative, clinical stage 2A (pT2pN1M0). In November 2010, a conservative surgery and axillary lymph node dissection were performed, and the patient underwent adjuvant chemotherapy (CT) with six cycles of Cyclophosphamide, Methotrexate, and Fluorouracil (CMF), followed by whole breast radiation therapy, and tamoxifen for 3 years suspended due to side effects.

In December 2019, an ipsilateral breast mastectomy was performed due to secondary ipsilateral *in situ* ductal carcinoma (grade 3, HR-positive). Patient refused further adjuvant therapy.

In December 2020, the patient was concerned about recent abdominal pain. A CT-scan revealed enlarged axillary lymph nodes and multiple liver metastasis. A lymph node biopsy confirmed the presence of a HR-positive and HER2-negative BC recurrence.

Considering that the patient had a 7-year treatment-free interval and without visceral crisis criteria, first-line treatment selection was letrozole plus abemaciclib. In April 2021, after 4 months of treatment, lymph node response and liver disease progression were observed. Considering the observed primary hormone resistance according to European School of Oncology- European Society for Medical Oncology (ESO-ESMO) 2016 Consensus Guidelines, and the rapid visceral progression, combined chemotherapy was selected with docetaxel plus capecitabine [1].

The clinical case was presented in our Molecular Tumour Board (MTB) and two formal recommendations were done: a liver biopsy to retest HR and HER2 status by immuno-histochemistry (IHC), and its evaluation using the next-generation sequencing (NGS) FoundationOne CDx (F1CDx) platform [2].

IHC assay in the liver sample (LEICA BOND MAX®) revealed the presence of high oestrogen receptor expression (70%), absence of progesterone receptor and HER2 staining. A qualitative assay of 11 mutations of the PIK3CA gene (LRG_310t1) commercial kit AmoyDx with Cobas Z 480 (Roche) equipment was utilised to evaluate most frequent mutations, including H1047R, H1047Y y H1047L, E542K, E545K, E545D, E545A, E545G, Q546R, Q546E in exon 9 and C420R in exon 7. No somatic genetic alterations were identified.

In June 2021, after two cycles of chemotherapy the patient experienced new liver disease progression. At that time, the F1CDx assay informed the presence of the pathogenic variant N588fs*26 in BRCA2 gene. More findings are detailed in Table 1.

Taking in account the patient’s clinical course and the F1CDx results, the MTB second session discussion recommended genetic counseling for germline testing and to start systemic therapy with talazoparib.

After genetic counseling (Figure 1), a 33-gene panel from Invitae (San Francisco, CA) including genes related to colon, gynecological and colorectal cancer was performed (APC, ATM, AXIN2, BARD1, BMPR1A, BRCA1, BRCA2, BRIP1, CDH1, CHEK2, DICER1, EPCAM, GREM1, MLH1, MSH2, MSH3, MSH6, MUTYH, NBN, NF1, NTHL1, PALB2, PMS2, POLD1, POLE, PTEN, RAD50, RAD51C, RAD51D, SMAD4, SMARCA4, STK11, TP53). None of the evaluated genes demonstrated any relevant mutation or variation.

**Figure 2.**
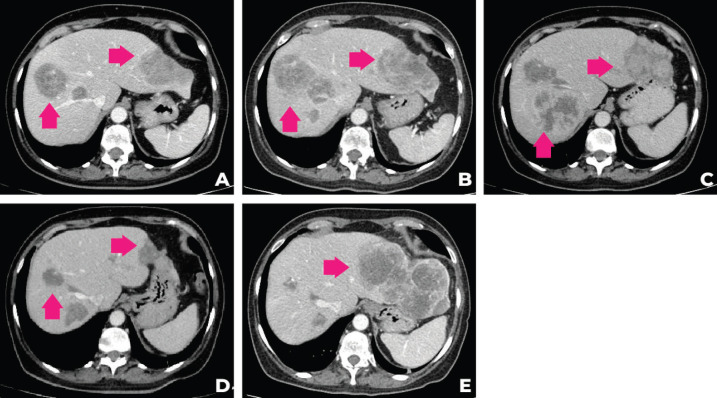
Evolution of the patient’s computer tomography scans in different time periods. (a): CT of December 2020, (b): April 2021, (c): June 2021, (d): September 2021, (e):December 2021.

**Figure 3.**
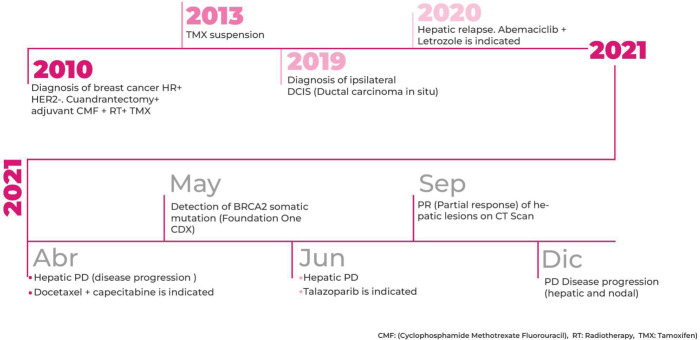
Timeline of main patient treatments and outcomes.

In September 2021, after 3 months of talazoparib, clinical improvement of abdominal pain and liver partial response was observed. Tumour response was ongoing at the time of this case submission (December 2021). A summary of the patient’s main radiologic findings is portrayed in Figure 2 and a timeline of patient evolution in Figure 3.

## Case discussion

NGS has proven to be a key implementation to understand biological pathways involved in cancer. In daily practice, the identification of somatic and germline mutations has allowed physicians to gather relevant information to make therapeutic decisions and benefit patients [3]. Importantly, somatic mutations provide targeted opportunities for treatment and reveal resistance mechanisms to understand patients’ evolution. In this context, somatic mutations in BRCA1/2 (sBRCA1/2m) and other homologous recombination related genes (HRRG) alterations are observed in nearly 10% of tumour samples [4].

Our case reflected the case of a patient that had poor response to classic therapeutic strategies for HR+/HER2− ABC, and NGS led to the finding of a sBRCA1/2m.

Scanty data in clinical trials and in real world setting is available regarding utility of PARPi in pathogenic or likely-pathogenic sBRCA1/2m and/or germline or somatic HRRG mutations (g/sHRRGm) in ABC.

Tung *et al* [5] evaluated the efficacy of olaparib in a single-arm phase II study in patients with HER2-negative ABC with sBRCA1/2m or g/sHRRGm other than BRCA1/2. In the group of 16 patients that had sBRCA1/2, the objective response rate (ORR) was 50% with a median progression free survival (PFS) of 6.3 months (90% confidence interval (CI), 4.4 months-not available) [5]. In the subgroup associated with gPALB2 mutations, the ORR was 82% with a median PFS of 13.3 months (90% CI, 12 months-not available). No responses were observed in patients with ATM or CHEK2 mutations alone.

In another phase II study, Gruber *et al* [6] reported the efficacy of talazoparib in patients with breast and non-breast cancer with g/sHRRGm. Thirteen patients with ABC were included. The authors observed a total ORR of 25% and a clinical benefit rate of 50% with this treatment [6].

Tumour mutation profiling can have germline implications. Particularly, the subgroup analysis of the OlympiAD study tissue samples from 161 out of 302 patients demonstrated a 99% of concordance between gBRCAm and tBRCAm.

In a retrospective series, Principe *et al* [7] identified 221 patients as having at least one pathogenic or likely pathogenic sBRCA1/2 alteration. 65% of them underwent germline genetic testing [7]. A total of 49% of the evaluated patients harboured gBRCA1/2m. Under these circumstances, it should be highlighted that the National Comprehensive Cancer Network (NCCN) guidelines recommend confirmatory germline genetic testing for any individual with sBRCA1/2 finding regardless of tumour type [8].

In this context, INTERCEPT was a multicentric prospective and observational study that evaluated the incidence of germline mutations in a cohort of 2,984 patients, using the 80-gene Invitae platform [9]. The authors concluded that around one in every eight patients with BC diagnosis had genetic variants associated with poor prognosis, and nearly in one third of included participants, proposed treatment strategy was modified according to NGS results. By this criteria and contrasting to the diagnostic sequence performed in our case, germline genetic assessment should be the first step to investigate possible prognostic and predictive factors.

Other studies have evaluated the implications of somatic gene sequencing to facilitate enrolment to clinical trials and define targeted treatment approaches.

In the SAFIR-01 study, André *et al* [10] examined the incidence of PIK3CA and AKT1 mutations, as well as other druggable amplifications in a cohort of 423 BC patients by comprehensive genomic hybridisation and Sanger sequencing. Among 297 patients with evaluable results, 46% had genomic alterations, being PI3KCA mutations (25%) and CCND1 (19%) and FGFR1 (13%) amplifications the most frequent findings. Targeted approaches were offered to 13% of study participants. ORR were only observed in four individuals included in the cohort.

The evidence of somatic targetable alterations in different tumour models have led to International Societies to evaluate indications for somatic sequencing technologies for different treatment scenarios. ESMO guidelines have established that multigene sequencing should not be routinely offered in patients with ABC [11].

Widespread access to NGS involves immense amounts of information and result interpretation becomes a challenge for the multidisciplinary team. The main objective is to distinguish between findings that represent proven or potentially relevant alterations, based on preliminary clinical or preclinical evidence.

The European Society for Medical Oncology (ESMO) proposes the ESMO Scale for Clinical Actionability of molecular Targets (ESCAT) scale, which provides a systematic framework for ranking molecular targets based on the available evidence supporting their value as therapeutic targets. It allows prioritisation of cancer genomic alterations with improved interpretation of NGS findings. Findings are classified as ‘levels I-C’ if clinical trials in multiple tumour types, or combined clinical trials, have demonstrated a clinically meaningful benefit in the specific tumour type [11].

To achieve this conclusion, authors classified commonly found somatic alterations in this population regarding actionability (ESCAT scale). BRCA1/2 somatic mutations are classified as ESCAT III, considering that clinical benefit was mainly observed in other tumour models [12].

In the recently presented clinical trial SAFIR-02, André *et al* [10] presented the results of 238 patients, who had clinical benefit with chemotherapy for ABC and were randomised to switch to an NGS-mutation matched targeted therapy or continue with maintenance chemotherapy. In the subgroup of 115 patients that had tumours with ESCAT I or II mutations – associated with proved or likely to predict benefit –, a significantly longer PFS was demonstrated in the arm that received targeted therapy (9.1 versus 2.8 months, respectively). Specifically, in cases with tumours with HRD score equal or over 42, PFS was 10.2 months with targeted therapies compared to a median of 2.7 months observed in patients that received maintenance chemotherapy [13].

Our case raises the question of when NGS should be considered. Under these circumstances, the role of MTBs is essential. To illustrate this, Hlevnjak *et al* [14] reported the experience of the first 200 ABC patients that had a DNA or RNA whole-genome or transcriptome sequencing (CATCH study). Of the 128 subjects that were discussed in a MTB and in 64 cases (50%), treatment was indicated considering MTB’s recommendations. Comparatively to the MOSCATO-01 study, the authors reported that 30% of those patients achieved a 1.3-fold PFS compared to the immediately prior therapeutic strategy PFS [15].

## Conclusions

Undoubtedly, more information regarding the cost effectiveness of NGS is needed to develop adequate reimbursement policies for this technology. Taking the lessons learnt in our case, our approach is to recommend NGS in HR+/HER2− ABC patients provided they have good performance status, when there is a lack of effective treatment options or when primary hormone resistance is evidenced. When appropriate, germline evaluations should always be recommended first.

Finally, it should be highlighted that the generalisation of MTBs and the implementation of molecular screening programmes are highly needed in our region to gain more knowledge of somatic mutations implicated in the Hispanic and Latin-American population with BC diagnosis. Hispanic patients, specifically those from low-income Latin American countries, are underrepresented in clinical trials in relation to their cancer incidence worldwide. Clinical trials leading to Food and Drug Administration (FDA) approval of new targeted treatments for BC have only included Hispanic patients in the range of 0%–9% [16]. Trials involving precision medicine studies for ABC, such as Andrew *et al* and Litton *et al* did not report the race or ethnicity of the participants [17, 18]. For those reasons, real-world studies that address these important question are essential in our population.

## Observations

Informed consent was obtained to publish personal information and images of the patient.

## Conflicts of interest

The authors declare that they have no relevant conflicts of interest regarding this publication.

## Funding

The authors of his work did not receive any specific grant from funding agencies in the public, commercial, or not-for-profit sectors.

## Figures and Tables

**Table 1.** NGS results of the liver sample analysis.

Gene	Protein effect	VAF	CNA
BRCA2	N588ffs*26	26.78%	—
KRAS	G12R	23.51%	—
ESR1	Amplification-equivocal	—	6
MCL1	E110del	57.21%	—
RB1	L220fs*3	24.94%	—
TP53	E224D	38.23%	—
